# First three-dimensional documentation of double-wave reentry in humans

**DOI:** 10.1016/j.hrcr.2021.03.011

**Published:** 2021-03-13

**Authors:** Philippe Maury, Maud Tabuteau, Franck Mandel, Hubert Delasnerie, Maxime Beneyto, Quentin Voglimacci-Stephanopoli, Anne Rollin

**Affiliations:** ∗ Department of Cardiology, University Hospital Rangueil, Toulouse, France; † I2MC, INSERM UMR 1297, Toulouse, France; ‡ Boston Scientific, Voisins le Bretonneux, France

**Keywords:** Ablation, Atrial tachycardia, Double-wave reentry, 3D mapping, Electroanatomical mapping, Reentry

## Introduction

Double-wave reentry (DWR) is an uncommon reentry mechanism where 2 independent waves are circulating in the same circuit at the same time, doubling the rate of activation. Such mechanism had been postulated from experimental findings, but clinical demonstrations remain limited. We report the first 3D documentation of clinical DWR in a patient with atrial tachycardia (AT).

## Case report

A 69-year-old man was referred for a new ablation procedure for AT recurring after 2 previous percutaneous ablation procedures for atrial fibrillation. He had undergone mitral and aortic valve replacements 12 years ago, together with concomitant surgical atrial fibrillation ablation.

During the previous ablation procedure, multiple fractionated areas and complex and various right and left ATs were observed, with complete pulmonary vein isolation and a full set of complete linear lesion block at the end of the procedure (roofline, cavotricuspid isthmus, and mitral isthmus line).

**Figure 1:**
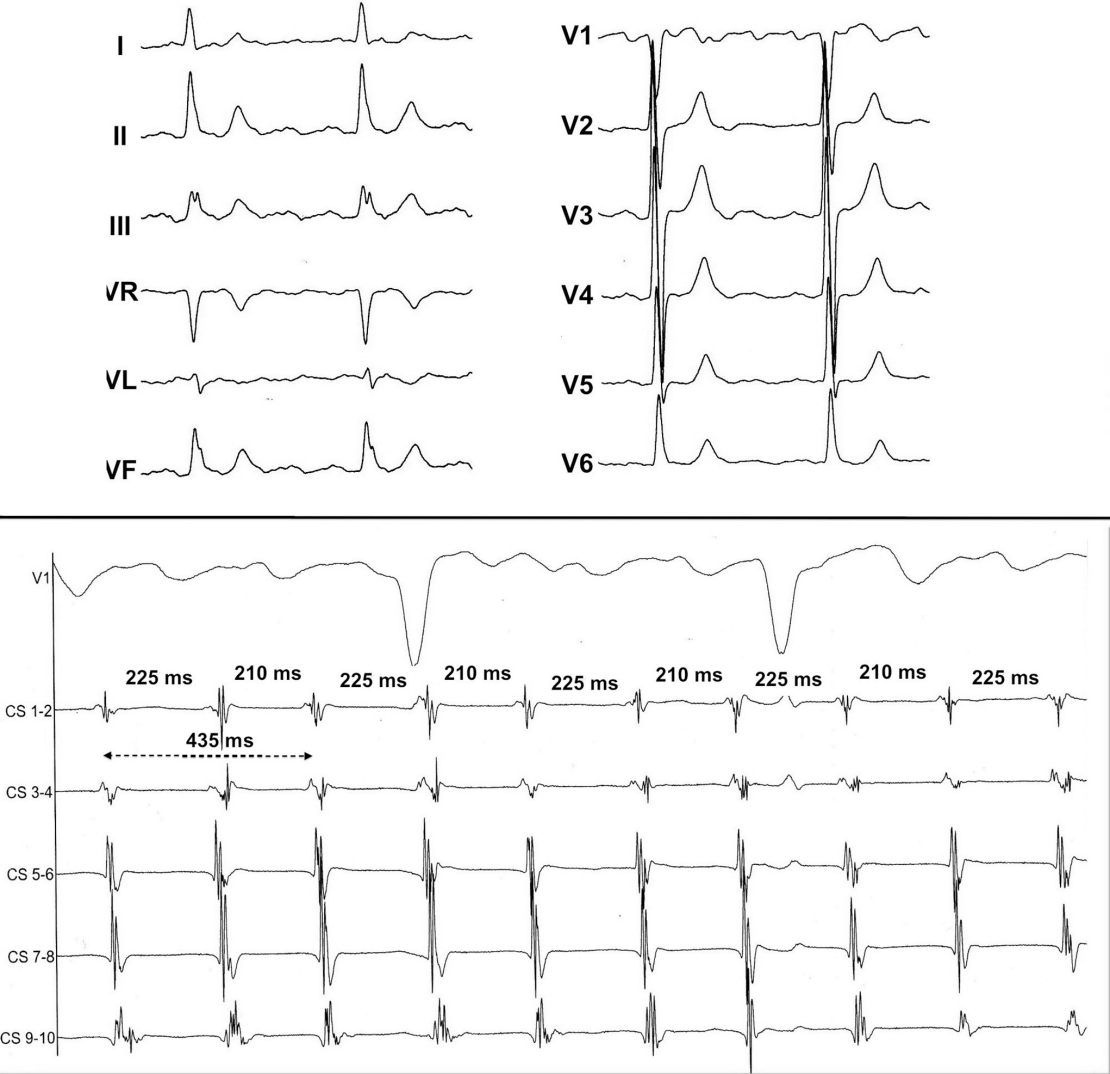
Twelve-lead electrocardiogram of the atrial tachycardia (upper) and recordings at the coronary sinus showing clockwise circuit around the mitral annulus, from distal to proximal recordings, with cycle length alternans (lower).

**Figure 2:**
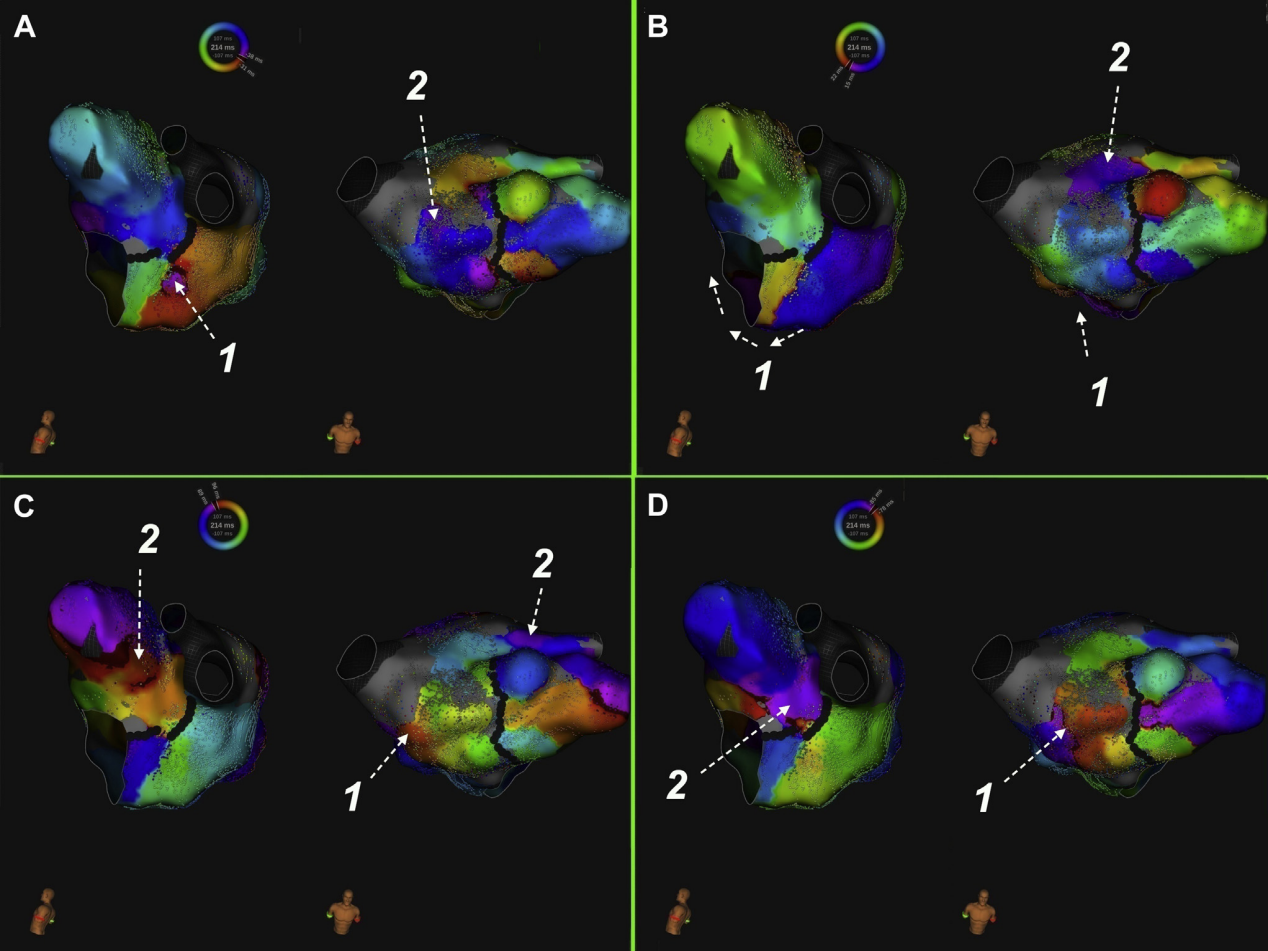
Successive panels showing the propagation of waves 1 and 2 in left lateral and anterior views during the apparent cycle length of the tachycardia. There were 2 different waves circulating in the same circuit and full completion of activation of each wavefront lasted twice the apparent cycle length. In the Rhythmia™ system (Boston Scientific, Marlborough, MA), the wavefront is depicted in brown/violet colors. **A:** Wave 1 is arising at the mitral isthmus below an apparent line of block, while wave 2 is located at the superior aspect of the left atrial septum. **B:** Wave 1 is turning inferiorly around the mitral annulus, while wave 2 is getting superiorly and posteriorly towards the ridge. **C:** Wave 2 is now descending the ridge, while wave 1 is ascending the left atrial septum. **D:** Wave 2 is apparently blocked at the mitral isthmus line, while wave 1 is still progressing towards the superior aspect of the left atrial septum. Thus during 1 apparent single atrial tachycardia cycle, wave 1 was wandering over half of the circuit while wave 2 activated the other half, replacing wave 1 at the mitral isthmus line after panel D. Note that the activation wavefront spent around 130 ms at the superior aspect of the left atrium (between panels A and C), representing the area of slow conduction of the circuit.

**Figure 3:**
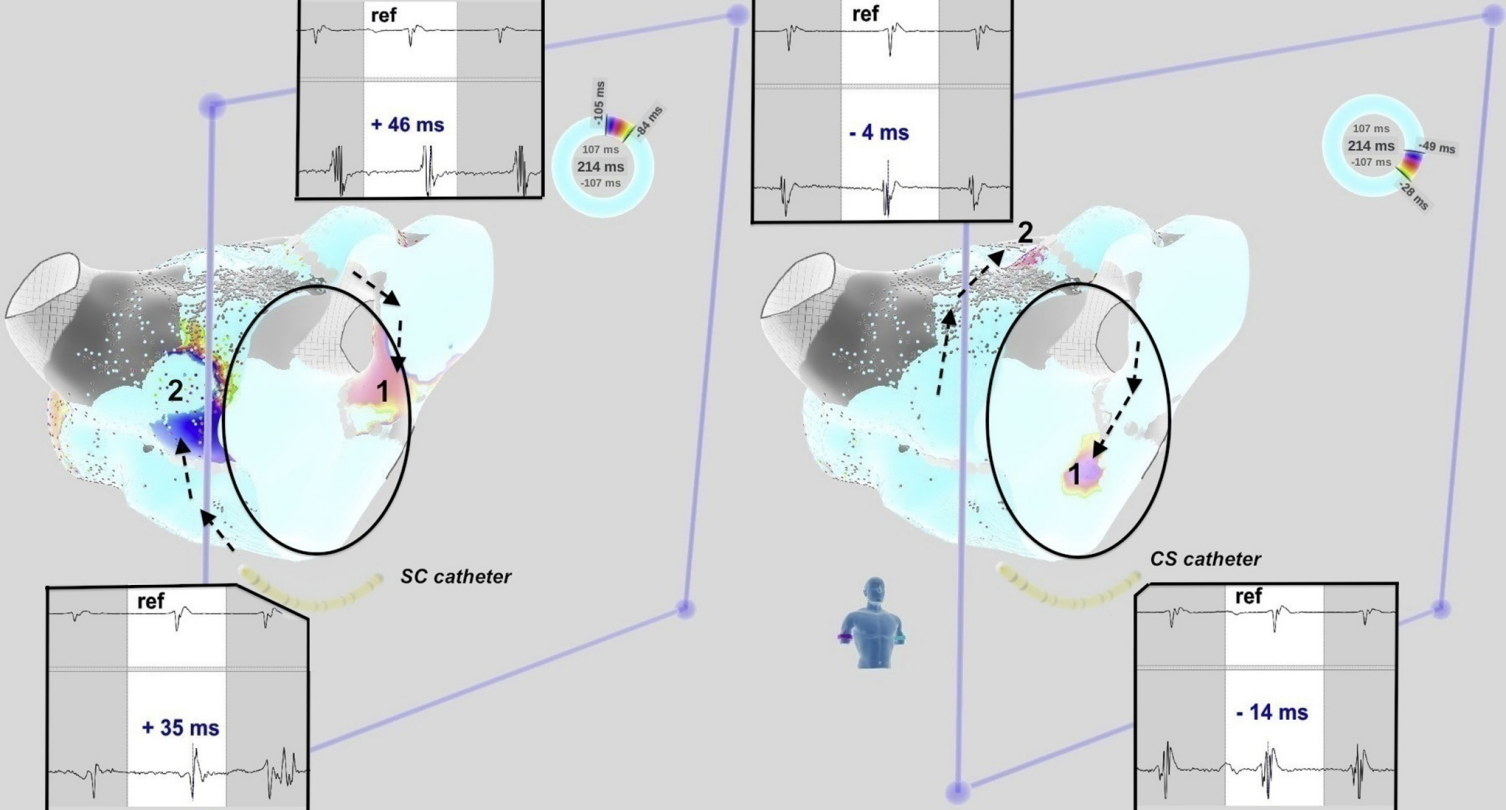
Anterior view of the left atrium (with clipping plane at the mitral annulus) showing the 2 wavefronts turning around the mitral annulus at the same time. Reference is recorded on the catheter located inside the coronary sinus. Local electrograms are shown, demonstrating that simultaneous activations at opposite sides of the circuit were present: simultaneous anterior and posterior and simultaneous superior and inferior activations regarding the same reference timing.

During the current procedure, performed off-drug except for beta-blockers, an irregular AT could be mapped using the Rhythmia™ system (Boston Scientific, Marlborough, MA), showing 210 and 225 ms alternating cycle lengths at the reference catheter located inside the coronary sinus (Figure 1). The mapping window was set to the apparent averaged cycle length. A complex activation rotating over the mitral annulus was observed. When carefully looking at the wave propagation, it appears that 2 independent activation wavefronts were circulating at the same time over the same perimitral circuit. Each wave followed the same unique circuit; there was no dual loop or 2:1 bystander activation elsewhere. The waves were arising from a possible “epicardial” component—ie, endocardial breakthrough just below the apparently blocked mitral isthmus—then turning onto the low septal side before wandering between areas of block at the superior aspect of the left atrium and then descending on the ridge before finally arising again on the lower aspect of the mitral isthmus line (Figures 2 and 3). Changing the mapping window from the short to the long AT cycle did not significantly change the activation pattern. Simultaneous activations at opposite sites of the circuit were present (ie, simultaneous anterior and posterior and simultaneous superior and inferior activations regarding the same reference timing) (Figure 3 and Supplemental Movie).

Each wave displayed a whole duration of activation of 435 ms and was detected once by the reference catheter, thus doubling the rate of activation. The alternating 210 and 225 ms cycle lengths were caused by the nonequidistant location of each wave into the circuit (short cycle length because of the second wave being short-coupled to the first one, and the converse), and the sum equaled 435 ms—ie, the duration of the full revolution of each wave. No entrainment or extrastimuli was performed.

Radiofrequency ablation at the complex fragmented septal areas and at the mitral isthmus endocardial breakthrough changed the tachycardia into double-loop reentry (roof and mitral isthmus dependent AT), then to focal AT arising from the ridge, and finally to common atrial flutter, which could all be successfully ablated. No AT recurred during the 2 years of follow-up.

## Discussion

To our knowledge this is the first 3D documentation of DWR. Although DWR was demonstrated 20 years ago after detailed analysis of multielectrode mapping as a potential mechanism for some ventricular tachycardia (VT) or atrial flutter, 3D activation maps of DWR had not been presented before.

DWR was induced in experimental models of VT with long cycle length and large excitable gap,^1^, ^2^, ^3^, ^4^ with a cycle length representing 50%–70% of the single-wave cycle length.^1^^,^^2^ DWR may happen when an extrastimulus is blocked close to the pacing site, so it is not colliding antidromically with the next arriving wave while being conducted in the orthodromic direction, thus creating a new wave inside the circuit.^1^^,^^4^^,^^5^ Both waves travel through partially refractory tissues with subsequent decrease of conduction velocity,^3^ and the wavelength should be shorter than half the size of the circuit.^2^^,^^3^ Class III drugs hinder and class 1 favor induction of DWR.^2^^,^^6^ Experimentally, DWR was the mechanism in 12% of sustained monomorphic VT^5^ and the cause of all VT accelerations during entrainment.^4^^,^^5^ A high degree of acceleration (<70% of the initial cycle length) with the same QRS morphology would suggest DWR as the mechanism for VT acceleration^1^^,^^2^ and may explain some proarrhythmic effects of class 1 drugs. Cycle length alternans can be observed because the 2 waves may not be evenly located within the circuit^4^; thus DWR may be a potential mechanism for cycle length alternation during VT. However, to date there is no available demonstration of ventricular DWR in clinical practice.

In humans, transient DWR could have been observed in half of cases during common atrial flutter after delivering a single premature stimulus at the cavotricuspid isthmus shortly after the end of the refractory period.^6^ The ratio of DWR cycle length to the baseline flutter cycle length ranges from 60% to 75%, with beat-to-beat variations^6^ for the same aforementioned reasons (such as in our case). DWR was affirmed by simultaneous occurrence of local activation at 2 opposite anatomic sites with an identical activation sequence and electrocardiogram morphology, as during spontaneous typical flutter.^6^ In opposition to the previous description of induced transient atrial DWR by Cheng,^6^ the stability and natural occurrence of this arrhythmia and alternans in our case was probably caused by the dilated left atrium and atrial conduction disturbances (eg, at the superior aspect of the left atrium; Figure 2), lengthening the reentry circuit and increasing the excitable gap. DWR has also been described during acceleration of atrioventricular reciprocating tachycardia by fast ventricular pacing, with rate-dependent left bundle branch block and atrioventricular node conduction delay increasing the excitable gap and the size of the circuit.^7^

DWR means that 2 independent waves are circulating in the same circuit at the same time, doubling the rate of atrial or ventricular activation. DWR cannot be easily mapped using current 3D electroanatomical systems, since these are unable to take into account that the true cycle length is double what is recorded and that there are 2 instead of 1 activation to be annotated when the window of mapping would be thus extended. No 3D system can annotate a point twice in a given window of mapping; thus this window is restricted to the apparent cycle length, which does not represent the true duration of revolution of each wave, but rather the duration between successive waves. Simple visualization of propagation while keeping the window of mapping close to the apparent cycle length—as especially well performed in the Rhythmia system—allowed us to reveal DWR as a succession of 2 independent rotating wavefronts around the mitral annulus over the same circuit. Using the Lumipoint™ algorithm,^8^ we were able to more clearly depict the existence of the double wave (Supplemental Figure 1). Ripple mapping may be another means to better visualize this mechanism on the CARTO™ system. Otherwise, in 3D mapping systems, DWR can be viewed as a focal tachycardia with a cycle length being half of the duration of the whole propagation over the circuit.^9^ During the window of mapping—corresponding to the apparent cycle length and to half of the revolution time—another wave could be visualized, propagating along the same direction at the opposite site of the circuit, corresponding to the other concomitant wave-forming DWR.

## Appendix Supplementary data

Supplemental Movie Loop of the double-wave reentry.

Supplemental Figure 1 Visualization of the double wave using the Lumipoint™ algorithm. (The Lumipoint algorithm automatically highlights areas with ongoing depolarization at a given period of time, irrespective of color-coded timing seen in standard activation maps.)
