# Supplementary material for: First three-dimensional documentation of double-wave reentry in humans
Source: HeartRhythm Case Rep. 2021 Mar 13;7(6):374–7. doi: 10.1016/j.hrcr.2021.03.011 (PMC8226329; doi:10.1016/j.hrcr.2021.03.011)
Supplement: Supplemental Figure 1 — Visualization of the double wave using the Lumipoint™ algorithm. (The Lumipoint algorithm automatically highlights areas with ongoing depolarization at a given period of time, irrespective of color-coded timing seen in standard activation maps.) [file mmc2.doc]

Supplementary material:

**Supplementary Figure 1:** visualization of the double wave using the Lumipoint **™** algorithm (The Lumipoint **™** algorithm automatically highlights areas with ongoing depolarization at a given period of time, irrespective of colour-coded timing seen in standard activation maps)

**Movie:**  loop of the double wave reentry
