# Supplementary figures and images for: First three-dimensional documentation of double-wave reentry in humans
Source: HeartRhythm Case Rep. 2021 Mar 13;7(6):374–7. doi: 10.1016/j.hrcr.2021.03.011 (PMC8226329; doi:10.1016/j.hrcr.2021.03.011)

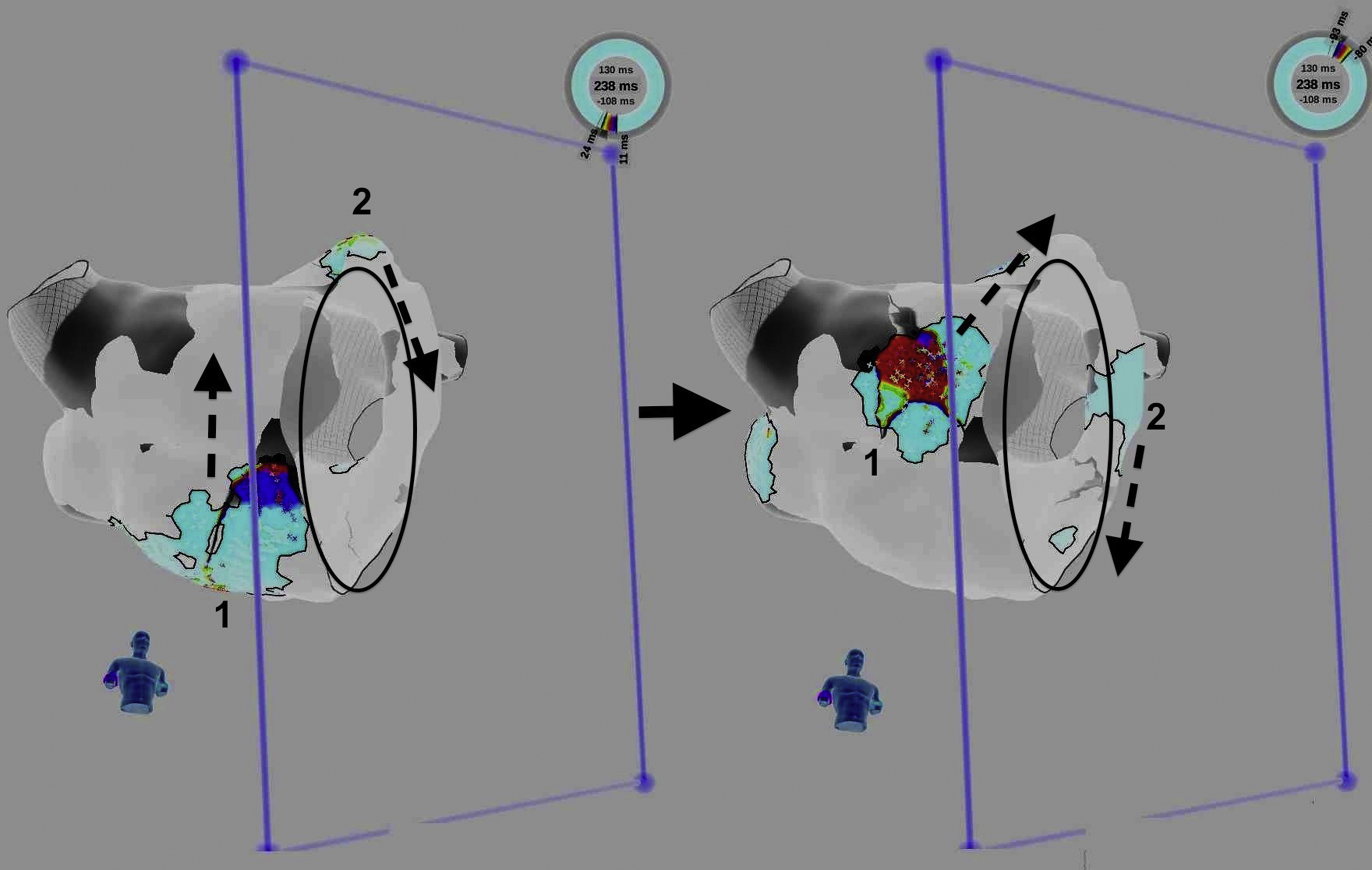

Supplement: Supplementary File [file figs1.jpg]
